# Artificial neural network-based analysis of ferroptosis-associated molecular subtypes and immunological profiles in abdominal aortic aneurysm

**DOI:** 10.3389/fimmu.2026.1721069

**Published:** 2026-02-02

**Authors:** Maohua Li, Shasha Xiao, Qi Qin, Keyun Fu, Lunchang Wang, Xin Li, Jiehua Li

**Affiliations:** 1 Department of Vascular Surgery, The Second Xiangya Hospital of Central South University, Changsha, China; 2 Molecular Biology Research Center, School of Life Sciences, Hunan Province Key Laboratory of Basic and Applied Hematology, Central South University, Changsha, China; 3 Institute of Vascular Diseases, Central South University, Changsha, China

**Keywords:** abdominal aortic aneurysm, artificial neural network, ferroptosis, immune microenvironment, molecular subtypes

## Abstract

**Background:**

Abdominal aortic aneurysm (AAA) is a severe vascular disease that can lead to rupture and life-threatening hemorrhage. The role of ferroptosis in AAA pathogenesis remains insufficiently understood. This study aims to investigate the role of ferroptosis in AAA by identifying ferroptosis-associated molecular subtypes and examining their relationship with immunological characteristics using an artificial neural network (ANN) model.

**Methods:**

We analyzed three publicly available datasets (GSE7084, GSE47472, and GSE57691) to identify differentially expressed ferroptosis-related genes (FRGs) and employed consensus clustering to classify AAA samples into two subtypes. Immune infiltration was assessed with the CIBERSORT algorithm, and a diagnostic artificial neural network (ANN) model based on subtype-specific genes was developed to discriminate ferroptosis-associated molecular subtypes and derive the NeuraAAA score.

**Results:**

Nine differentially expressed FRGs were identified, and the model incorporated three key genes (oncostatin M, heme oxygenase-1, and interleukin-6), achieving high diagnostic accuracy (AUC = 0.988). Consensus clustering stratified AAA samples into two ferroptosis-associated subtypes with distinct immune profiles, with the C1 subtype showing higher immune infiltration and immune scores than C2. The derived NeuraAAA score was elevated in the immune-enriched subtype and correlated with immune-cell infiltration, and a nomogram integrating NeuraAAA and immune score showed good calibration. Immunofluorescence confirmed increased expression of all three genes in AAA specimens.

**Conclusion:**

Our study reveals the heterogeneous role of ferroptosis in AAA pathogenesis, demonstrating that ferroptosis-associated subtypes are linked to variations in the immune microenvironment. These findings provide new insights into AAA pathophysiology and suggest potential targets for subtype-specific therapeutic strategies, contributing to advances in precision medicine for AAA treatment.

## Introduction

Abdominal aortic aneurysm (AAA) is a vascular disorder characterized by localized dilation of the abdominal aorta, which can lead to rupture and cause life-threatening hemorrhage. AAA rupture accounts for approximately 170,000 deaths annually worldwide, which primarily affects males over 65 (1, 2). The global prevalence of AAA is estimated to be 0.92% among individuals aged 30 to 79, with smoking, male sex, hypertension, and advanced age identified as major risk factors (3). Despite advances in surgical techniques like open surgery and endovascular aortic repair (EVAR), aneurysm-related deaths increased significantly, rising by 81.6% from 1990 to 2019 (4). There are currently no effective medical treatments to slow the advancement of AAA, which emphasizes the need for a better comprehension of molecular pathways to create new strategies to prevent AAA growth and rupture (1, 5).

There have been some reports that ferroptosis plays a critical role in AAA formation. Neutrophil extracellular traps (NETs) trigger ferroptosis by inducing oxidative stress, accelerating vascular smooth muscle cell (SMC) damage, and promoting AAA progression (6, 7). Mitochondrial glutathione (mitoGSH) is pivotal in regulating ferroptosis, with SLC25A11, a mitochondrial glutathione transporter, influencing NET-induced ferroptosis by modulating mitoGSH levels. NETs destabilize SLC25A11, leading to mitoGSH depletion and triggering ferroptosis in SMCs (6, 8). Additionally, dysregulated iron metabolism exacerbates ferroptosis in AAA, with ganglioside GM3 interacting with transferrin receptor 1 (TFR1) to increase iron uptake and oxidative stress (9). The SLC7A11/GPX4 axis, a primary antioxidant pathway, inhibits ferroptosis by suppressing lipid peroxidation. Ferroptosis inhibitors, such as Ferrostatin-1, have shown efficacy in slowing AAA progression in experimental models (10). Ferroptosis also promotes inflammatory responses in AAA via NF-κB signaling, while inhibiting ferroptosis or NF-κB signaling can significantly alleviate SMC damage and retard AAA progression (11). Therefore, molecules like SLC25A11, SLC7A11/GPX4, and GM3 emerge as potential therapeutic targets, offering new possibilities for targeted AAA treatments. Despite progress, the understanding of ferroptosis in AAA remains limited, particularly in identifying AAA-specific molecular subtypes and exploring ferroptosis’s role in the immune microenvironment. These insights may guide the development of targeted therapies for AAA.

In this study, ferroptosis-related genes (FRGs) that are differently expressed in AAA are comprehensively evaluated, and their relationship to the immunological microenvironment in AAA patients is clarified. The analysis of nine differentially expressed FRGs identified two distinct ferroptosis-associated subtypes, suggesting a heterogeneous role of ferroptosis in AAA pathogenesis. We developed an artificial neural network (ANN) model to support molecular subtype classification and provide additional insights into ferroptosis- and immunity-associated patterns in AAA. Immunofluorescence staining further supported the clinical relevance of ferroptosis in AAA by confirming increased expression of key subtype-associated genes in patient tissues. These findings establish a foundation for future therapeutic strategies targeting ferroptosis in AAA, with the potential to improve patient outcomes through precision medicine.

## Materials and methods

### Ferroptosis−related gene datasets and microarray data

Four AAA datasets (GSE7084, GSE47472, GSE57691 and GSE166676) (12–15)were obtained from the GEO database (https://www.ncbi.nlm.nih.gov/geo/). To eliminate batch effects and integrate these datasets, the SVA package was applied (16). Batch effect correction using the ComBat algorithm was performed on log2-transformed expression data. The GSE7084 dataset consists of sequencing data from 9 AAA patient samples and 10 normal aortic samples. The GSE47472 dataset includes sequencing data from 14 AAA neck samples and 8 donor aortic samples, with all samples classified as the control group. The GSE57691 dataset contains sequencing data from 49 AAA patient samples and 10 donor aortic samples. The GSE166676 is a single cell sequencing data from 3 AAA patient samples and 2 normal samples. Additionally, 394 human ferroptosis-related genes (FRGs) were retrieved from the FerrDb V2 database (http://www.zhounan.org/ferrdb/). Differentially expressed genes (DEGs) were identified using the limma package (17). Differentially expressed FRGs were identified with a screening threshold of |log2(fold change) | > 0.7 and p-value < 0.05. For subtype-specific DEGs, a more stringent threshold of |log2(fold change) | > 1 and p-value < 0.05 was applied.

### Single-cell RNA sequencing analysis

Single-cell RNA sequencing (scRNA-seq) data were analyzed using the Seurat R package. After quality control, normalization, and identification of highly variable genes, dimensionality reduction was performed by principal component analysis (PCA). Cell neighborhoods were constructed using the top principal components (dims = 1:30), followed by unsupervised clustering and cell-type annotation based on canonical marker genes. The t-distributed stochastic neighbor embedding (t-SNE) was used for visualization of cellular relationships. A composite NeuraAAA score, comprising OSM, HMOX1, and IL6, was calculated at the single-cell level using the AddModuleScore function in Seurat. The distribution of the NeuraAAA score across cell populations was visualized on the t-SNE embedding using FeaturePlot.

### Consensus clustering analysis

Based on the expression profiles of 9 FRGs, 58 AAA samples were clustered into distinct subtypes using the R package ‘ConsensusClusterPlus’ (18). Consensus clustering was performed with 50 iterations, each utilizing 80% of the samples, to stratify the AAA cases into clusters. The optimal number of clusters was established by evaluating the cumulative distribution function curves and the consistency observed in the matrix heatmap.

### Immune infiltration analysis

The CIBERSORT algorithm was applied to assess the relative abundance of 22 immune cell types in AAA from gene expression data (19). Only samples with CIBERSORT’s p-values < 0.05 were retained for downstream analyses. Furthermore, Spearman correlation analysis was conducted to examine the relationship between hub genes and immune cells.

### Construction of a diagnostic model based on artificial neural network machine learning

Least absolute shrinkage and selection operator (LASSO) regression was implemented using the glmnet package. Predictors were automatically standardized prior to model fitting, and an L1 penalty was applied (α = 1). The optimal regularization parameter (λ) was determined by 10-fold cross-validation, and model coefficients were extracted at the value of λ that minimized the cross-validated error (λ_min).Support vector machine (SVM) analysis was performed using the caret framework with a radial basis function kernel. Feature selection was conducted via recursive feature elimination (RFE) using all candidate features with cross-validation.

Based on the expression profiles of selected variables, we built a neural network model with 58 AAA samples using the R package ‘neuralnet.’ The dataset was randomly split into a training set (70%, N = 40) and a validation set (30%, N = 18). The artificial neural network (ANN) included a single hidden layer with 5 neurons. The model was trained on the training set to predict two output variables, C1 and C2. After training, the model’s performance was evaluated on the validation set. The R package “pROC” was used to evaluate classification performance and plot ROC curves. Finally, important gene weights were used to compute a disease classification score (NeuraAAA): Σ(weight_i × expression_i). Based on the average NeuraAAA score, the 58 AAA samples were divided into high- and low-score groups.

### Construction evaluation of a nomogram model

A nomogram model combining NeuraAAA and immunescore data was created using the R package “rms” to assess the likelihood of AAA subtypes. In this model, each factor is assigned a specific score based on its impact on the outcome, with the “total score” representing the sum of individual factor scores. The total score enables comprehensive risk assessment by integrating multiple variables into one predictive measure. Model performance was assessed using calibration curves and decision curve analysis (DCA). Calibration curves were used to compare predicted probabilities with observed outcomes, while DCA was applied to quantify the clinical net benefit across a range of threshold probabilities.

### Immunofluorescence staining

Human AAA specimens and paired, macroscopically non-aneurysmal proximal aortic neck control tissues were collected from six male patients undergoing elective open AAA repair (clinical characteristics in **Supplementary Table S1**). The study was approved by the Institutional Review Board at the Second Xiangya Hospital of Central South University (Protocol No.: LYEC2024-K0153) following the Helsinki Declaration, with patient consent obtained. Tissues were fixed in 4% paraformaldehyde, paraffin−embedded, and sectioned at 5μm. After deparaffinization, rehydration, and antigen retrieval, sections were incubated overnight at 4°C with primary antibodies against human Oncostatin M (OSM; ABclonal, A8705), Heme oxygenase−1 (HMOX-1; Proteintech, 10701−1−AP), or interleukin−6 (IL−6; Boster, BA4339), followed by appropriate fluorescent secondary antibodies and DAPI counterstaining.

For quantification, all images for a given target were acquired in one session using an Olympus BX51 fluorescence microscope with a 20× objective and identical exposure/gain settings. An investigator blinded to sample identity analyzed the images using ImageJ software (NIH). Five non−overlapping fields from three sections per patient were evaluated for each target. The mean fluorescence intensity (MFI) of the target channel was measured within the aortic media/adventitia region of interest (ROI). Background MFI (from an adjacent acellular area) was subtracted, and the resulting value was normalized to the DAPI−positive nuclear area within the same ROI. Normalized MFI values from all fields per patient were averaged to obtain a single value per patient per target. Statistical comparison between AAA and paired neck control groups was performed on these per−patient averages using GraphPad Prism.

### Statistical analysis

All statistical analyses and visualizations were performed using R (v4.2.1) and GraphPad Prism (v8.0.1). Differences between subgroups were assessed using either a t-test or Wilcoxon test, with statistical significance set at P < 0.05 unless otherwise specified.

## Results

### Differential expression analysis of ferroptosis-related genes in AAA

**Figure 1:**
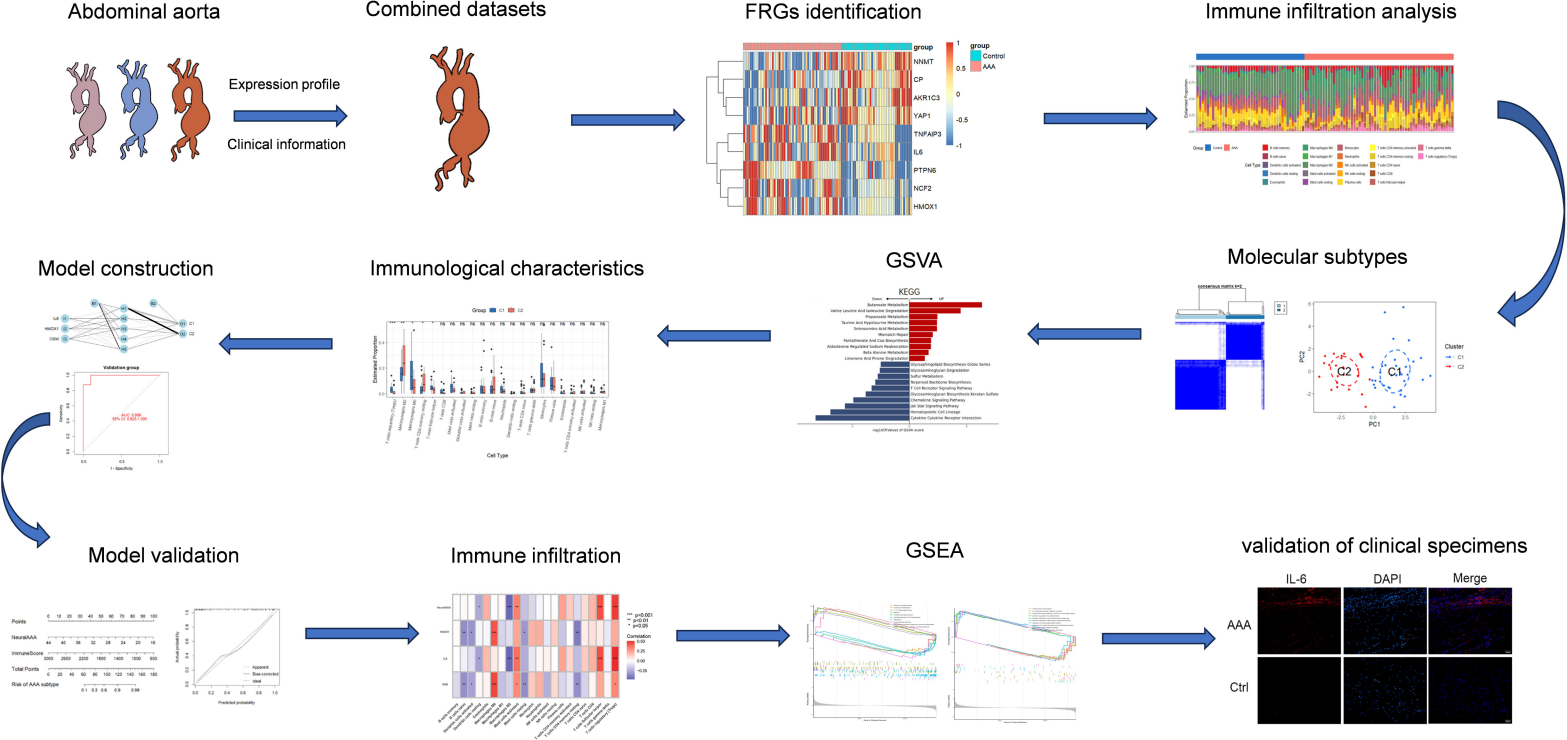
Flow diagram for this research.

**Figure 2:**
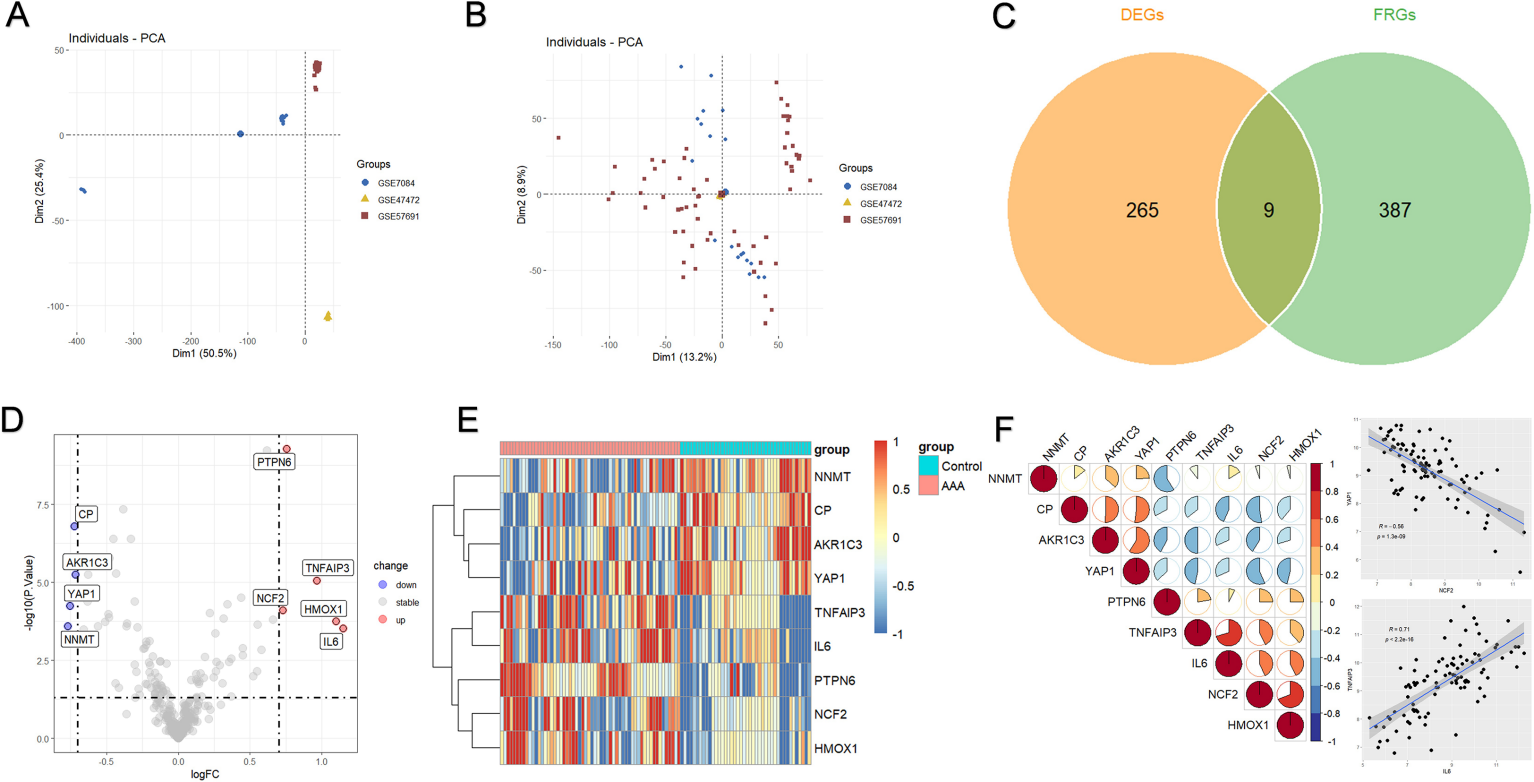
Ferroptosis regulators that are differently expressed in AAA patients. **(A, B)** GSE7084, GSE47472, and GSE57691 expression profiles are displayed in PCA plot both before **(A)** and after **(B)** batch effect adjustment. **(C)** Nine ferroptosis-related genes (FRGs) that are differently expressed in AAA patients are depicted in the Venn diagram. **(D)** A volcano graphic showing the differences in FRGs mRNA expression levels between AAA patients and normal control. **(E)** FRGs that are expressed differently in these groups are displayed in a heatmap. **(F)** Correlation plot of nine differentially expressed FRGs. The scatter plot depicts the two genes with the strongest positive and two genes with the strongest negative correlations.

**Figure 3:**
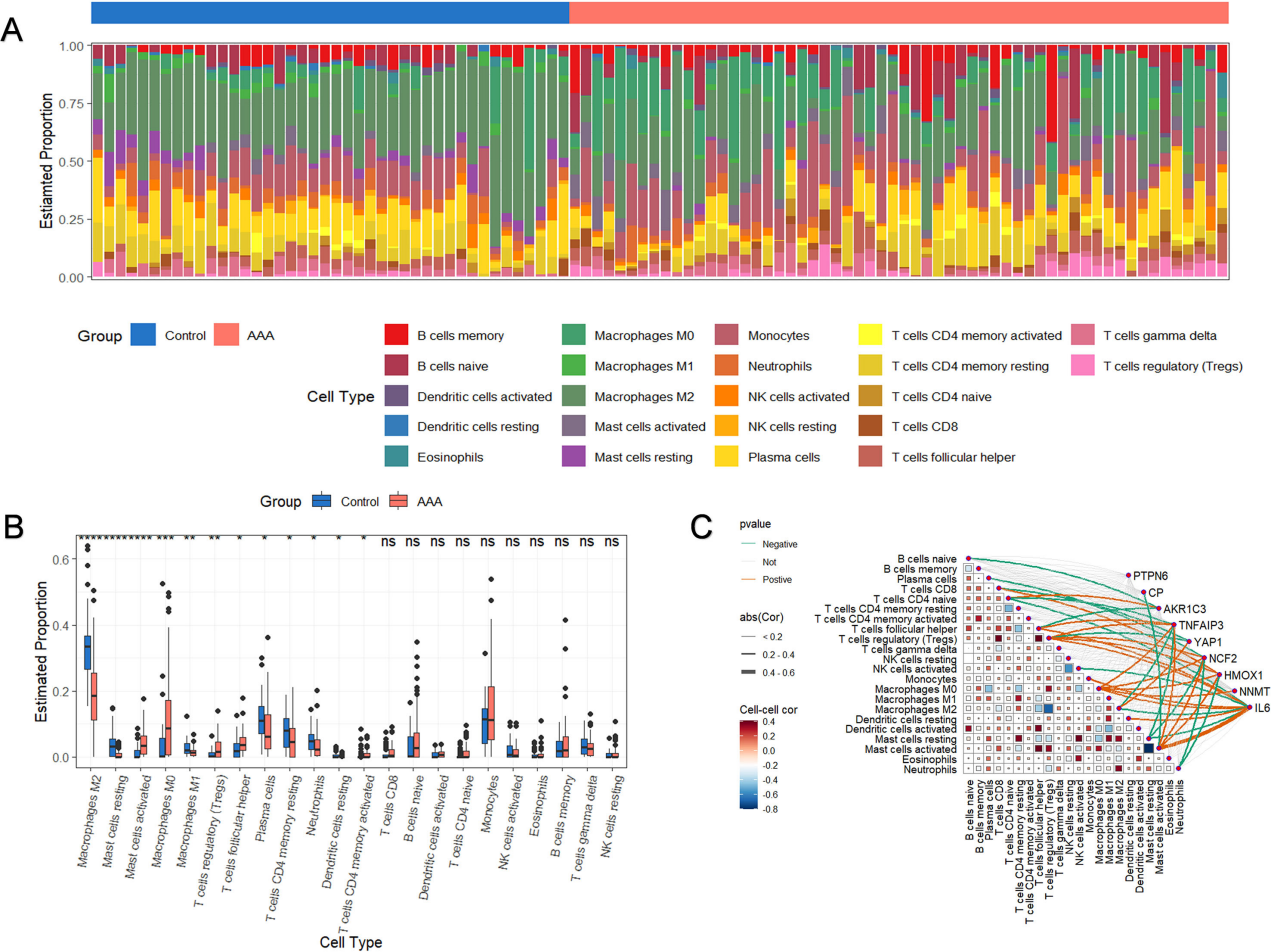
Immunological traits of AAA patients and control subjects, as well as associations between immune cells and distinctive ferroptosis-related genes (FRGs). **(A)** The relative amounts of 22 infiltrated immune cells in the control and AAA samples are displayed in a stacked chart. **(B)** Box plot showing the changes of infiltrated immune cells between control and AAA groups (*p<0.05, **p<0.01, ***p<0.001, ****p<0.0001). **(C)** Correlation network plot showing the correlations between immune cells and between immune cells and FRGs ns, no significance.

We analyzed the gene expression data from three datasets (GSE7084, GSE47472, and GSE57691) to identify differentially expressed ferroptosis-related genes (FRGs) in AAA. **Figure 1** provides a comprehensive flowchart of the investigation process. Principal Component Analysis (PCA) was used to visualize the expression profiles of these datasets. Prior to batch correction, PCA showed that PC1 and PC2 explained 50.5% and 25.4% of the total variance (**Figure 2A**), respectively, and samples were clearly separated according to dataset origin, indicating substantial batch effects. After ComBat correction, the variance explained by PC1 and PC2 was reduced to 13.2% and 8.9% (**Figure 2B**), respectively, and samples from different datasets exhibited substantial overlap, suggesting effective removal of batch effects. To explore the role of ferroptosis regulators in AAA progression, we initially identified 374 differentially expressed genes (DEGs) associated with AAA. By intersecting these DEGs with ferroptosis-related genes, we identified nine differentially expressed ferroptosis regulators (**Figure 2C**). Among them, PTPN6, NCF2, TNFAIP3, HMOX1, and IL6 were significantly upregulated, while CP, AKR1C3, YAP1, and NNMT were downregulated in AAA (**Figures 2D, E**). Spearman correlation analysis on these ferroptosis-related DEGs revealed strong inter-gene associations. Correlation scatter plots showed the strongest positive association between TNFAIP3 and IL6, and the strongest negative correlation between NCF2 and YAP1 (**Figure 2F**). Gene expression data were used to assess the relative proportions of 22 infiltrated immune cell types (**Figure 3A**). The findings showed that follicular helper T cells, regulatory T cells (Tregs), activated mast cells, and CD4+ T cell infiltration were significantly higher in AAA, suggesting that AAA development is associated with immune response alterations (**Figure 3B**). We conducted a correlation study to explore the association between ferroptosis-related genes (FRGs) and infiltrating immune cells in AAA. The results indicated significant associations between several immune cells and the nine differentially expressed FRGs, with notable interactions among immune cells themselves (**Figure 3C**). These results suggest that ferroptosis-related regulators are associated with immune cell infiltration patterns in AAA.

### Identification of ferroptosis-associated subtypes in AAA

**Figure 4:**
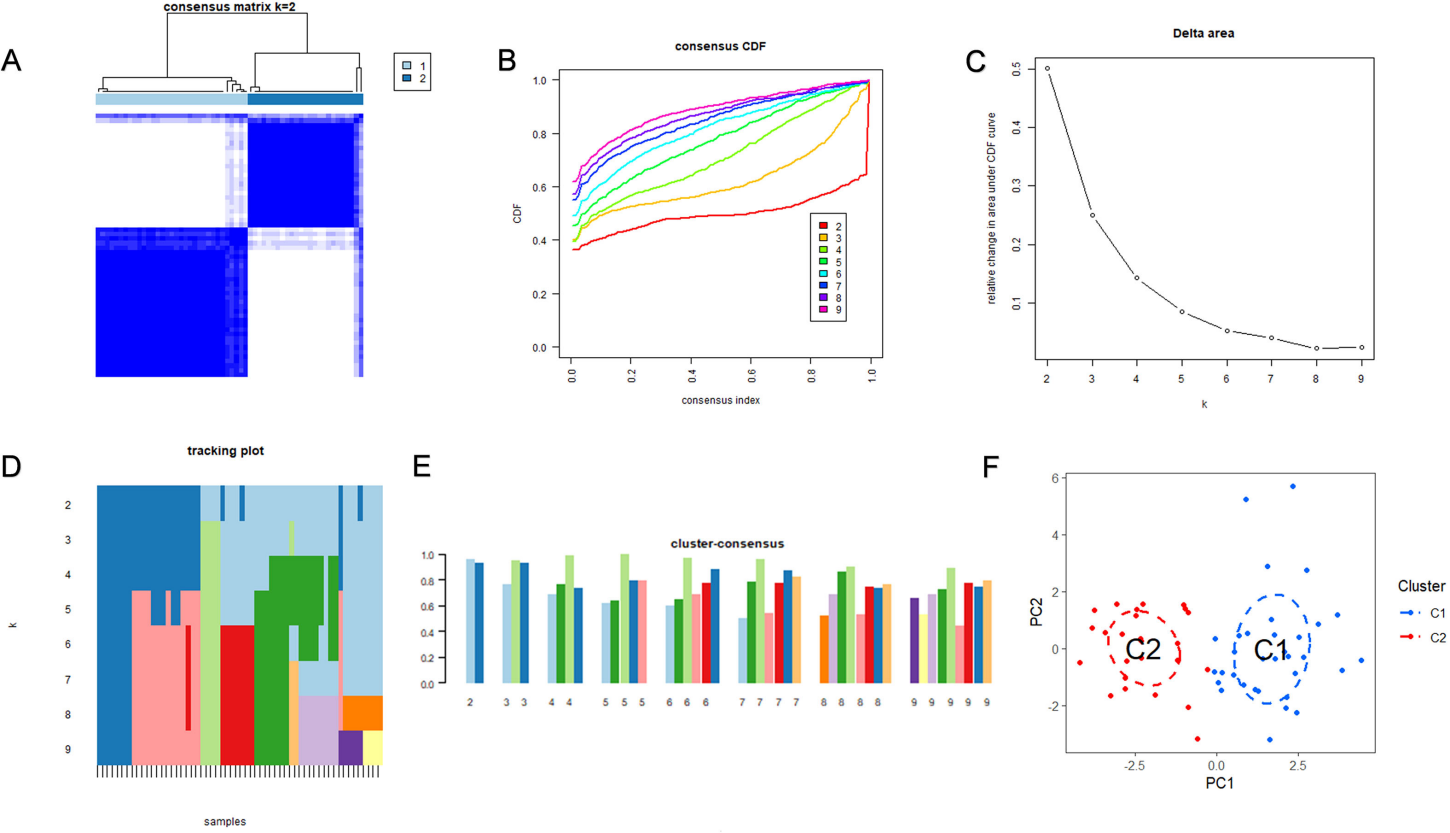
Molecular subtypes are identified using key ferroptosis-related genes (FRGs). **(A)** AAA patient consensus matrix (k=2), where samples are represented by rows and columns. White (zero) and dark blue (one) are the extremes of the consistency matrix. **(B)** The cumulative distribution function (CDF) of consensus for k = 2–9. **(C)** CDF curve delta area under the curve. **(D)** A tracking plot that displays each sample’s distribution among groups for k = 2 to 9. **(E)** The subtype score for k = 2–9 is represented by the consensus clustering score. **(F)** Two distinctive ferroptosis subtypes were identified in AAA patients using principal component analysis (PCA).

We performed unsupervised clustering on 58 AAA samples based on the expression profiles of nine differentially expressed ferroptosis-related genes (FRGs) to examine ferroptosis-related patterns in AAA. The consensus heatmap was interpreted such that values close to dark blue indicate that a pair of samples consistently clustered together across resampling iterations, whereas values close to white indicate low clustering consistency. At k = 2, the consensus matrix showed clear block-diagonal structures with sharp boundaries, indicating high within-cluster consensus and low between-cluster ambiguity (**Figure 4A**). Next, we calculated the proportion of ambiguous clustering (PAC) using a consensus index interval of 0.1–0.9. The PAC score was lowest at k = 2 (PAC = 0.190) compared with other clustering solutions (k = 3–9), indicating the lowest level of ambiguity and the highest clustering robustness (**Supplementary Table S2**). Third, additional stability measures, including cumulative distribution function (CDF) curves (**Figure 4B**), delta area plots (**Figure 4C**), tracking plots (**Figure 4D**), and cluster-consensus scores (**Figure 4E**), consistently supported k = 2 as the most stable and reproducible clustering solution. Consequently, the 58 AAA patients were divided into two ferroptosis-related subtypes, C1 (n = 33) and C2 (n = 25). The clear distinction between these two subtypes was verified by principal component analysis (PCA) (**Figure 4F**).

### Functional and pathway enrichment analysis of ferroptosis subtypes

**Figure 5:**
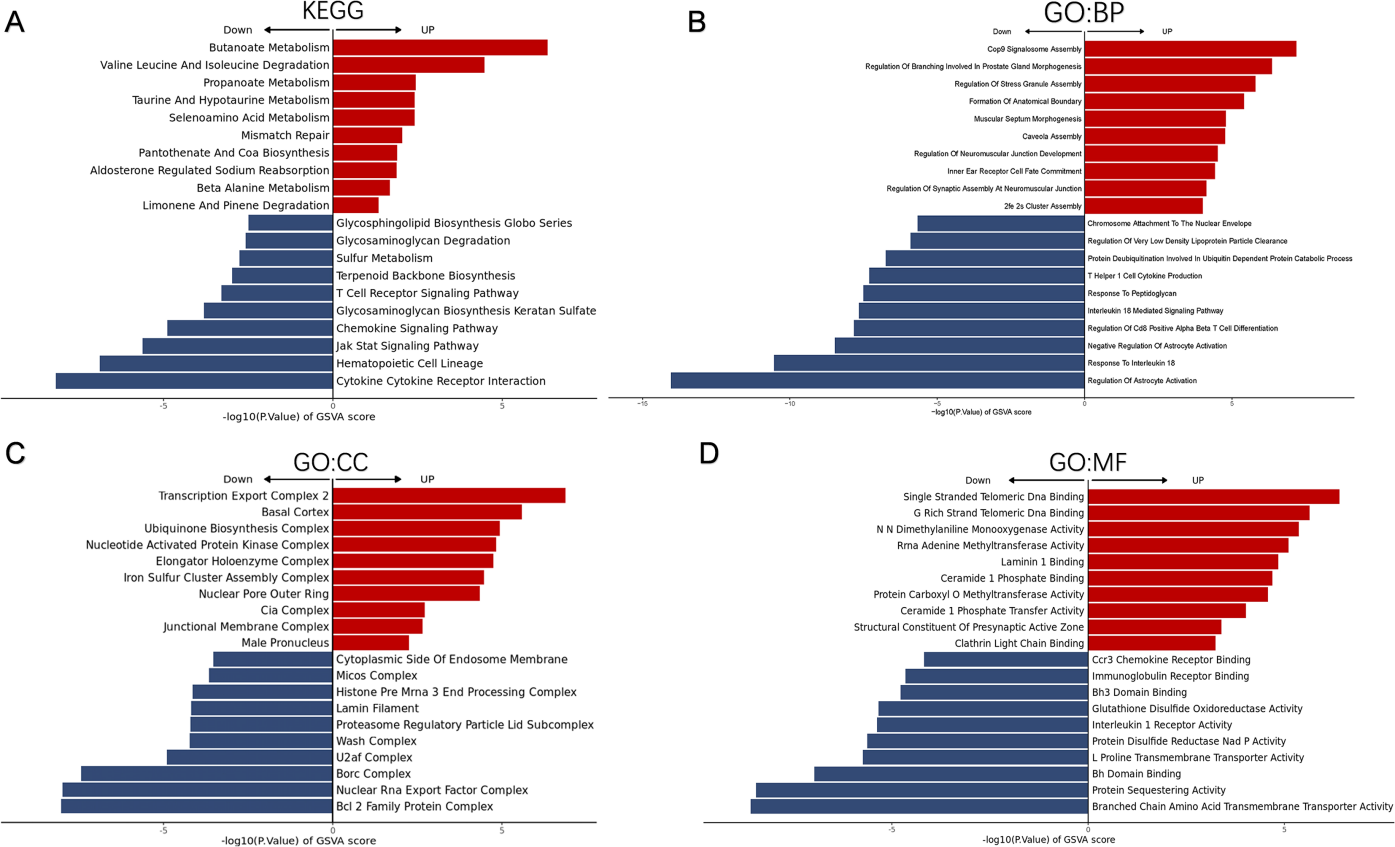
Differential biological, molecular, and cellular functions as well as signaling pathways between subtypes are identified. **(A-D)** The gene set variation analysis (GSVA) exhibits signaling pathways **(A)**, distinct biological processes **(B)**, distinct molecular functions **(C)** and distinct cellular components **(D)** between subtypes.

The gene set variation analysis (GSVA) was conducted to elucidate the biological roles of the two ferroptosis subtypes. Subtype C1 was associated with cytokine-cytokine receptor interactions, chemokine signaling, and T cell receptor signaling, while subtype C2 showed significantly higher enrichment in metabolic pathways. Functional enrichment analysis showed a significant association of subtype C2 with COP9 signalosome assembly, while subtype C1 was linked to T helper 1 cell cytokine production (**Figures 5A-D**). These findings suggest that ferroptosis subtype C1 is associated with a distinct immunological response profile.

### Identification of immune infiltration and immunological characteristics between ferroptosis subtypes

**Figure 6:**
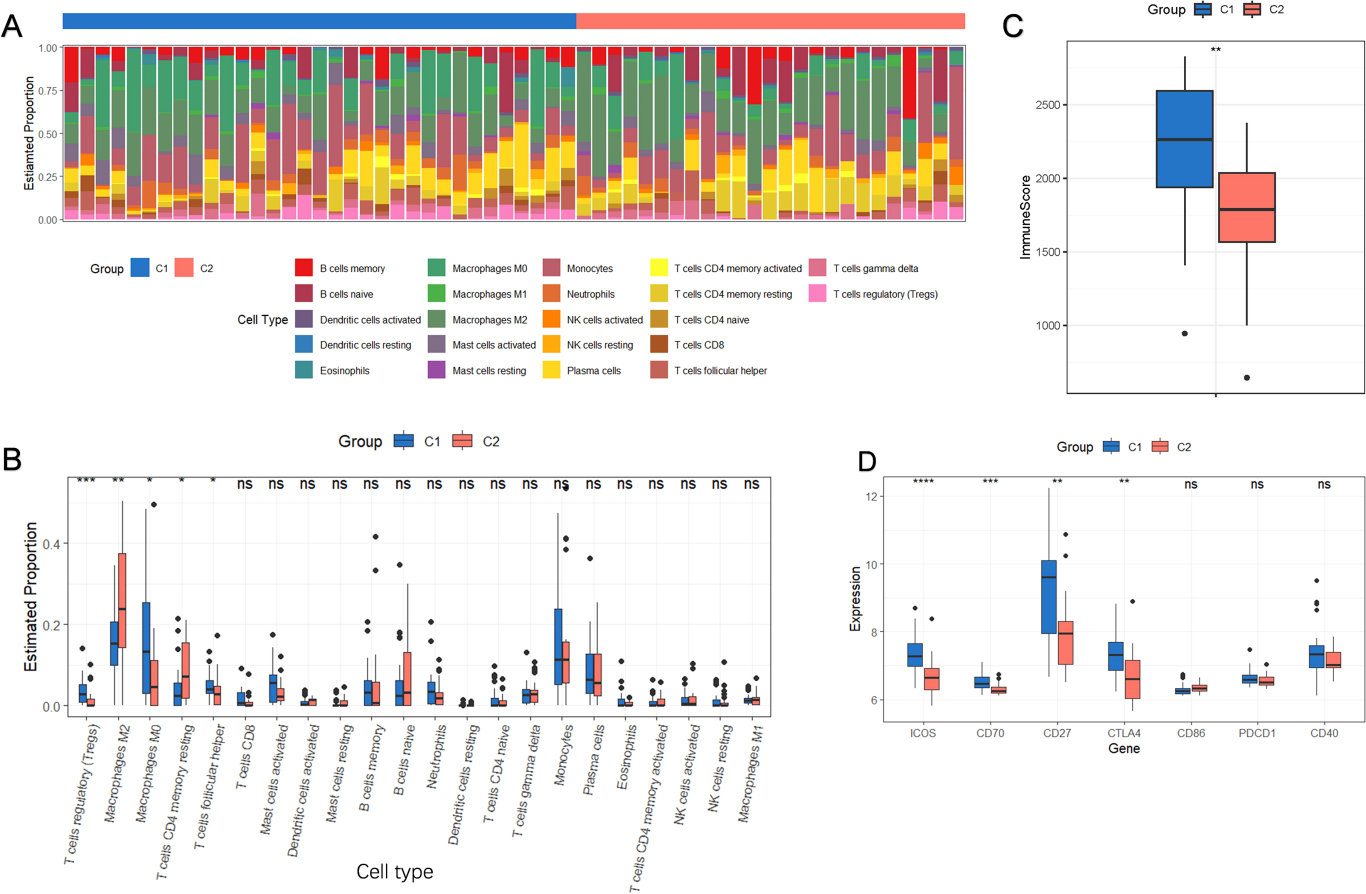
Correlation between ferroptosis subtypes and immune characteristics. **(A)** 22 immune cell subpopulation proportions in subtypes C1 and C2 are shown in a stacked bar chart. **(B)** Box plot showing the two ferroptosis subtypes’ relative immune cell infiltration abundances. **(C)** Box plot showing the differences in immune scores between the two ferroptosis subtypes. **(D)** Immune checkpoint mRNA expression levels between the two ferroptosis subtypes are displayed in box plot. #*p<0.05, **p<0.01, ***p<0.001. ns, no significance.

Immune cell infiltration was evaluated using the CIBERSORT method to investigate variations in the immunological microenvironment among ferroptosis subtypes. Significant differences in immune infiltration were observed between ferroptosis subtypes C1 and C2 (**Figure 6A**). Subtype C1 had a higher proportion of regulatory T cells, M0 macrophages, and follicular helper T cells, while subtype C2 exhibited significantly higher levels of M2 macrophages and resting memory CD4+ T cells (**Figure 6B**). The ESTIMATE algorithm was used to compute immune scores to further measure immune infiltration between the two subtypes. Subtype C1 consistently exhibited a higher immune score, indicating stronger immune infiltration (**Figure 6C**). Additionally, immune checkpoints and conventional immunological genes were assessed in AAA patients across all ferroptosis subtypes. Subtype C1 displayed significantly higher expression of immune checkpoint genes (ICOS, CD70, CD27, and CTLA4) than subtype C2 (**Figure 6D**), indicating a more pronounced immune-activated transcriptomic profile in subtype C1.

### Construction and evaluation of a predictive model

**Figure 7:**
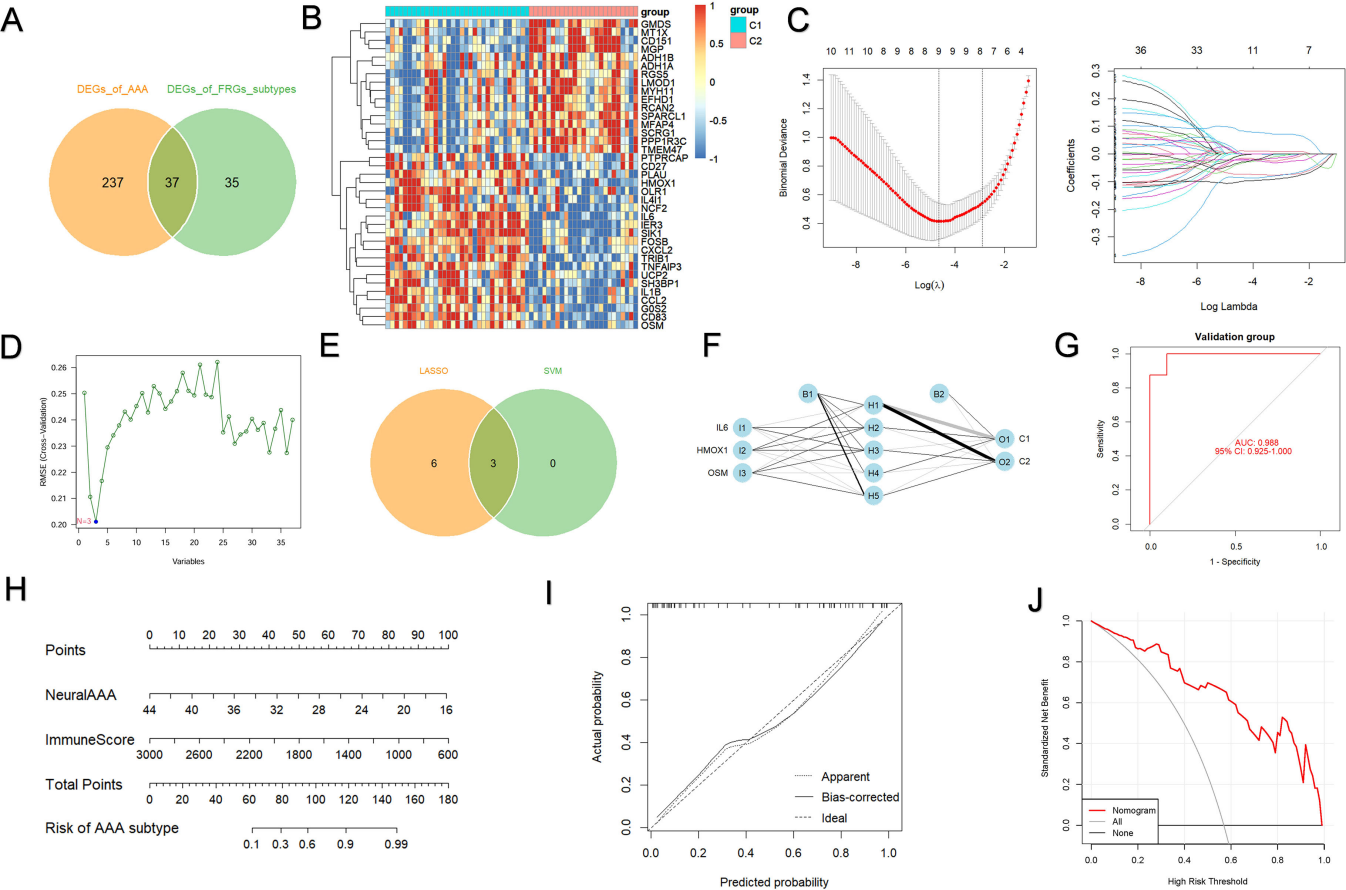
Construction and evaluation of a predictive model. **(A, B)** AAA patients’ 37 ferroptosis subtype-specific feature genes are displayed in the Venn diagram **(A)** and heatmap **(B)**. **(C)** Lasso regression identified 9 subtype characteristic genes. **(D)** SVM machine learning method identified 3 subtype characteristic genes. **(E)** Venn diagram displaying important genes related to ferroptosis subtype characteristics identified by both lasso regression and SVM. **(F)** Neural network machine learning model visualization. **(G)** Evaluation of the validation cohort’s classification performance using the neural network model. **(H)** A nomogram that predicts the progress of AAA by integrating NeuraAAA and immunological scores. **(I)** Calibration curves were employed to assess the diagnostic performance of the nomogram. **(J)** Decision curve analysis (DCA) curves comparing the clinical utility of the nomogram model.

**Figure 8:**
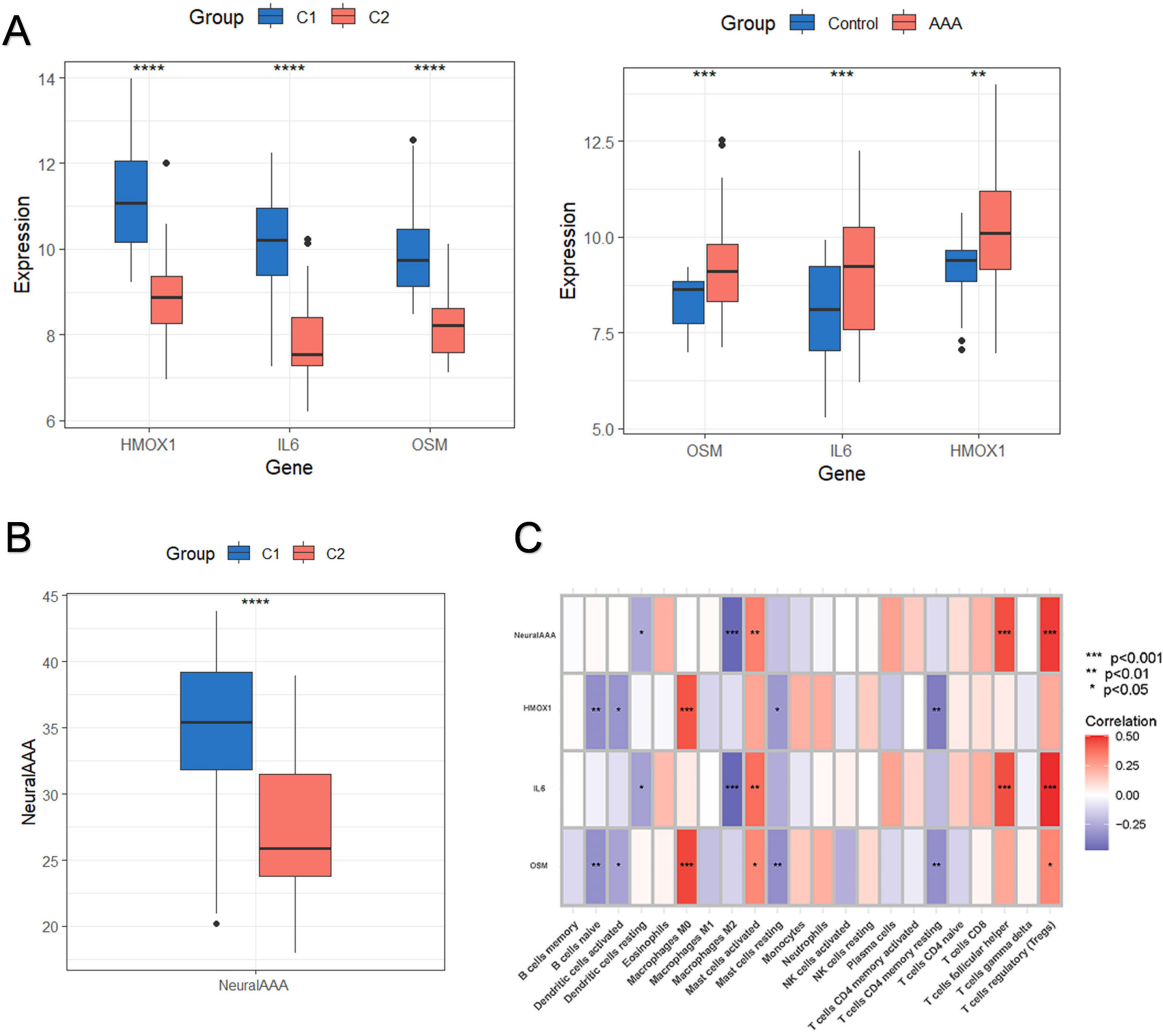
NeuraAAA scoring model and immune microenvironment association. **(A)** Box plot showing the three model genes’ expression profiles in relation to different subtypes and disease controls. **(B)** Box plots showing the differences in NeuraAAA scores between the C1 and C2 subtypes. **(C)** Heatmap showing the relationship between model genes, NeuraAAA, and distinct immune cell compositions (*p<0.05, **p<0.01, ***p<0.001, ****p<0.0001).

By crossing the AAA-related DEGs with the subtype-related DEGs, we were able to identify the subtype-specific DEGs and further validate the molecular subtypes based on FRGs (**Figure 7A**). A total of 37 subtype-specific DEGs were identified and visualized through a Venn diagram and heatmap to show their distribution and expression across subtypes. These genes were presented through a Venn diagram and heatmap to display their distribution and expression levels across different subtypes (**Figure 7B**). Lasso regression selected 9 key subtype-specific genes (**Figure 7C**), and support vector machine (SVM) analysis further refined the selection to 3 genes (**Figure 7D**). Results from both machine learning methods were combined in a Venn diagram, highlighting the common key genes: OSM, HMOX1 and IL6 (**Figure 7E**). A neural network-based machine learning model was constructed and visualized to predict AAA subtype characteristics (**Figure 7F**). The model’s classification performance was assessed in the validation cohort, achieving an area under the ROC curve of 0.988 (0.925-1), indicating high subtype discrimination ability (**Figure 7G**). Furthermore, A nomogram model based on NeuraAAA and immune scores calculated using the ESTIMATE algorithm was developed to estimate the probability of AAA subtype classification (**Figure 7H**). Calibration curves showed strong predictive performance and high diagnostic accuracy for the model (**Figure 7I**). Decision curve analysis (DCA) demonstrated that the nomogram consistently yielded a higher standardized net benefit than both the treat-all and treat-none strategies across a broad range of clinically relevant risk thresholds (**Figure 7J**). Correlation analyses further revealed that the NeuraAAA score was significantly and positively associated with KLF4 and ICAM1 (**Supplementary Figures S1A, B**). KLF4 is a well-established transcriptional regulator involved in smooth muscle cell (SMC) phenotypic switching and stress responses, whereas ICAM1 reflects inflammatory activation of SMCs. In the single-cell analysis, the NeuraAAA score was relatively higher in macrophages compared with other cell populations, indicating that this gene signature is more prominent within the macrophage compartment (**Supplementary Figures S1C, D**). We further explored the relationship between NeuraAAA and ferroptosis subtypes. Expression differences of the three signature genes among subtypes and control groups were analyzed using a box plot (**Figure 8A**), which showed that subtype C1 had a strong correlation with elevated NeuraAAA. The high-NeuraAAA group also had a comparatively higher immunological score (**Figure 8B**), suggesting a close association with immune responses. These findings indicate that high-NeuraAAA groups in AAA patients may predict ferroptosis subtypes and correlate with immunity. We examined the relationship between the three signature genes and significantly infiltrating immune cells, finding strong correlations with regulatory T cells, follicular helper T cells, and activated mast cells (**Figure 8C**).

### Distinct signaling pathways in high and low NeuraAAA score groups

**Figure 9:**
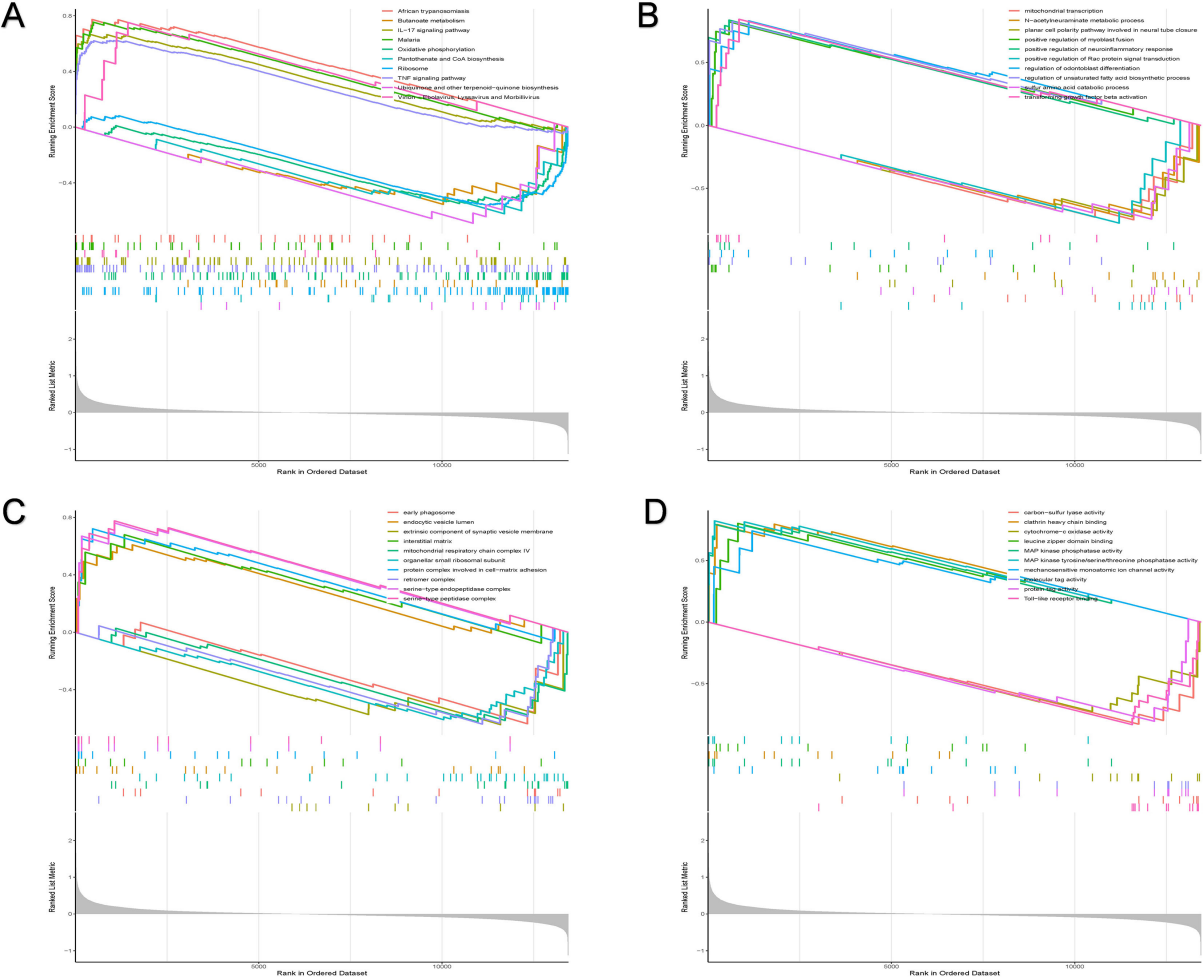
Characterization of the signaling pathways and biological functions that differ between the high and low NeuraAAA groups. **(A-D)** The gene set enrichment analysis (GSEA) displays distinct signaling pathways **(A)**, biological processes **(B)**, distinct molecular functions **(C)**, and distinct cellular components **(D)** between high-and low-NeuraAAA groups.

The gene set enrichment analysis (GSEA) was conducted to better understand functional differences between NeuraAAA groups. Results indicated distinct biological processes, signaling pathways, molecular activities, and cellular components between high- and low-NeuraAAA groups (**Figures 9A-D**). It was discovered that the following specific signaling pathways were significantly enriched in the high NeuraAAA group: KEGG_ IL-7_SIGNALING_PATHWAY, KEGG_TNF _ PATHWAY and KEGG_MALARIA (**Figure 9A**). Additionally, the following biological processes were primarily displayed by the high NeuraAAA group: TRANSFORMING_GROWTH_FACTOR_BETA_ACTIVATION, POSITIVE_REGULATION_OF_NEUROINFLAMMATORY_RESPONSE (**Figure 9B**). Moreover, the high-NeuraAAA group was characterized by the enrichment of the following molecular functions: ENDOCYTIC_VESICLE_LUMEN, PROTEIN_COMPLEX_INVOLVED_IN_CELL_MATRIX_ADHESION, RETROMER_COMPLEX, SERINE-TYPE_ENDOPEPTIDASE_COMPLEX (**Figure 9C**). In addition, the high-NeuraAAA group chiefly displayed the following cellular components: CLATHRIN_HEAVY_CHAIN_BINDING, LEUCINE_ZIPPER_DOMAIN_BINDING (**Figure 9D**). These findings suggest that immune activation is a primary characteristic of the high NeuraAAA group, indicating a closer association with inflammatory and immune-related processes in AAA.

### Validation of the expression of the three candidate biomarkers in clinical specimens

**Figure 10:**
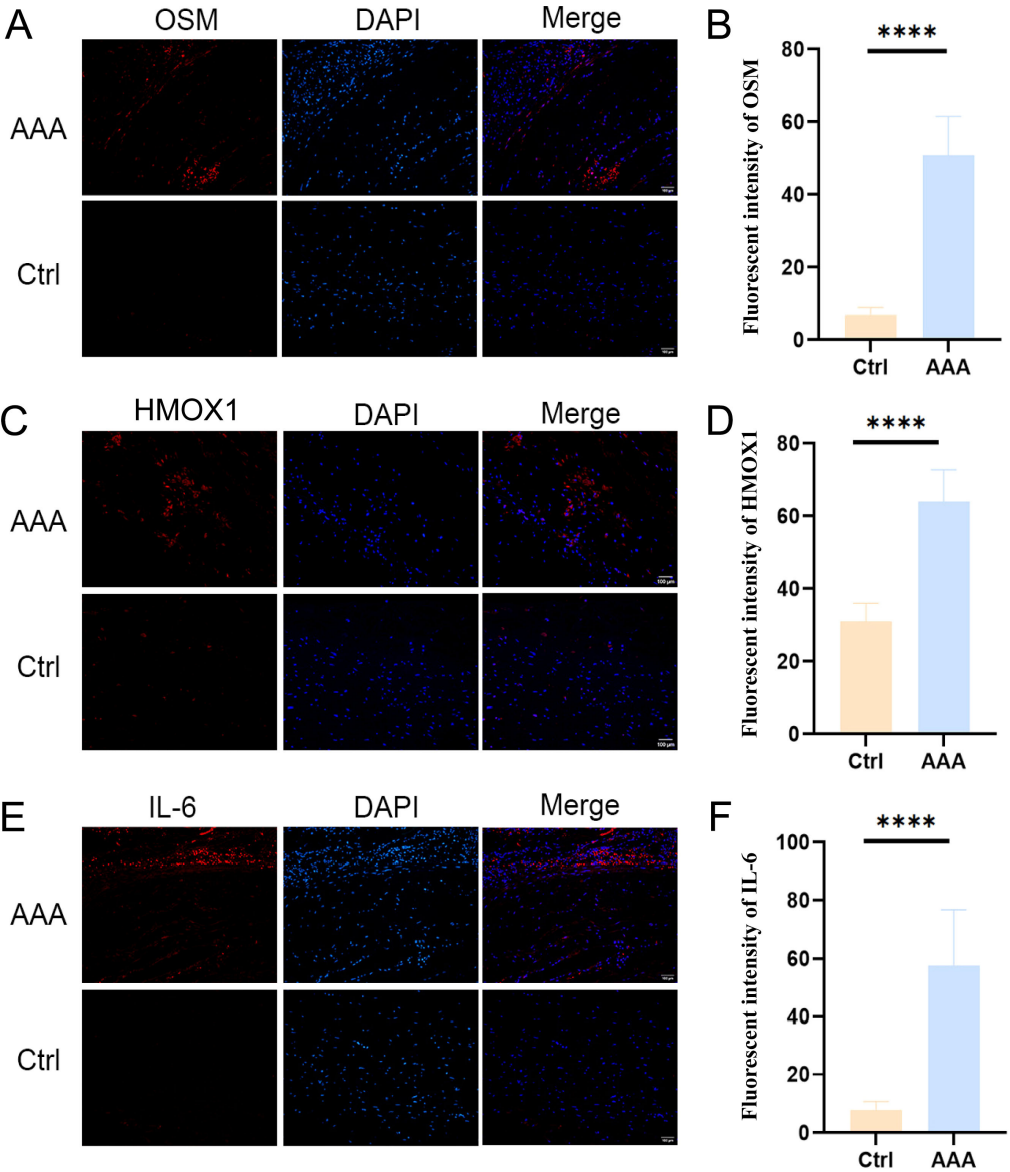
Validation of the expression of the three candidate biomarkers in clinical specimens of AAA with immunofluorescence (IF) staining. **(A B)** Relative expressions of Oncostatin M (OSM) in AAA tissues and aneurysm neck controls, as determined by IF techniques. **(C, D)** Relative expressions of Heme oxygenase-1 (HMOX-1) in AAA tissues and aneurysm neck controls, as determined by IF techniques. **(E, F)** Relative expressions of interleukin-6 (IL-6) in AAA tissues and aneurysm neck controls, as determined by IF techniques. Mean ± SEM, n = 6. ****P <0.0001.

Immunofluorescence (IF) staining was carried out in clinical specimens from AAA patients to verify the expression levels of three diagnostic biomarkers. IF staining showed significantly higher expressions of Oncostatin M (OSM) (**Figures 10A, B**), Heme oxygenase-1 (HMOX-1) (**Figures 10C, D**) and interleukin-6 (IL-6) (**Figures 10E, F**) in AAA tissues compared with aneurysm neck controls.

## Discussion

This study provides new insights into the pathophysiology of abdominal aortic aneurysm (AAA) by identifying ferroptosis-associated molecular subtypes and their immunological features through artificial neural network (ANN) learning. Our findings emphasize the crucial role in the pathophysiology of AAA and reveal the heterogeneity of this iron-dependent cell death mechanism and its interactions with the immune microenvironment. These discoveries offer a foundation for targeted therapeutic strategies focused on modulating ferroptosis and regulating the immune system in AAA management.

Ferroptosis, an iron-dependent form of cell death driven by lipid peroxidation, is implicated in diseases such as cancer, neurodegeneration, and inflammation. Its unique mechanism offers therapeutic potential, particularly for targeting drug-resistant cancers and inflammatory conditions (20–22). Ferroptosis is mediated through two main pathways: extrinsic (transporter-dependent) and intrinsic (enzyme-regulated). The core mechanism of ferroptosis involves an imbalance between oxidation and antioxidant systems, driven by abnormal redox enzyme activity. Lipoxygenase enzymes, such as ACSL4, promote lipid peroxidation, while the antioxidant enzyme GPX4, reliant on glutathione, acts as a major suppressor of ferroptosis. Ferroptosis can be regulated at various levels, including the transcriptional, post-translational, and epigenetic layers (23–25). Ferroptosis has also been implicated in the pathophysiology of various cardiovascular diseases, including heart failure, myocardial infarction, and atherosclerosis (26–28).

Our study identified two ferroptosis-related molecular subtypes of AAA that were tightly linked to distinct immune profiles. This association should be viewed as evidence of crosstalk rather than one-way causality, because directionality will require mechanistic validation. Notably, several ferroptosis-related genes (FRGs) highlighted in our analysis, including IL6, TNFAIP3, and NCF2, are well-recognized inflammatory regulators (29–31), and their enrichment likely reflects the crosstalk between ferroptotic stress and immune activation in the AAA microenvironment. We therefore propose a bidirectional, feed-forward loop: ferroptotic stress in vascular cells (especially smooth muscle cells) may amplify inflammation through damage-associated signals and lipid peroxidation products that activate pro-inflammatory pathways and promote immune-cell recruitment; conversely, an immune-enriched milieu may increase cytokine signaling and oxidative stress, disrupt iron and redox homeostasis, and impair antioxidant defenses, thereby sensitizing vascular cells to ferroptosis. Collectively, these transcriptomic patterns support an integrated ferroptosis–immunity axis in AAA pathogenesis rather than an exclusively ferroptotic or inflammatory program.

Based on three key FRGs (IL6, HMOX1, and OSM), the constructed ANN model provides a valuable tool that may aid in the molecular characterization and subclassification of AAA. IL-6 plays a crucial role in AAA development and progression through inflammatory pathways. Genetic polymorphisms in IL6 are linked to higher AAA risk, with specific IL6 genotypes correlating with increased inflammation and disease susceptibility (32). Elevated IL-6 levels contribute to inflammation within the aortic wall, activating signaling pathways such as STAT3, which promote cellular infiltration and disease progression (33). Experimental studies indicate although IL-6 has a protective role immediately following acute vascular injury, it perpetuates chronic inflammation, aiding in aneurysm growth (33). IL-6 neutralization studies suggest that suppressing IL-6 signaling can reduce AAA progression by limiting cellular infiltration and inflammatory response (34).

Heme oxygenase-1 (HMOX-1) degrades heme, reducing oxidative stress and inflammation by producing carbon monoxide (CO), biliverdin, and iron. HMOX-1 is crucial in protecting tissues from damage, especially in macrophages, where it limits inflammation in conditions like hemolytic disorders, sepsis, atherosclerosis, and metabolic syndrome. Its anti-inflammatory and antioxidant roles make it a potential therapeutic target for cardiovascular, metabolic, and inflammatory diseases (35, 36). HMOX-1 has been reported to play a protective role in AAA and resveratrol (RES) increases HMOX-1, which aids in reducing angiotensin II-induced vascular smooth muscle cell (VSMC) dysfunction (37).

Oncostatin M (OSM), a cytokine in the IL-6 family, is implicated in chronic inflammatory diseases, including inflammatory bowel disease (IBD) and multiple sclerosis (MS), as well as cardiovascular conditions like cardiac hypertrophy (38–40). Elevated OSM levels in IBD are linked to disease severity and anti-TNF drug resistance, suggesting that OSM may serve as both a therapeutic target and a biomarker for treatment-resistant patients. In MS, OSM is identified as a biomarker in both cerebrospinal fluid and plasma, aiding in diagnosis and disease monitoring. Additionally, in cardiovascular disease, OSM signaling through OSM receptor (OSMR) contributes to cardiac hypertrophy by promoting inflammation and impairing cardiac repair, indicating OSM as a potential target for heart failure treatment.

The ANN model achieved an area under the curve (AUC) of 0.988, indicating high performance in distinguishing ferroptosis-associated molecular subtypes and demonstrating the utility of machine learning in integrating multidimensional molecular data to support clinical assessment. Furthermore, the NeuraAAA score, which combines ferroptosis-related gene expression with immune infiltration features, provides a novel composite biomarker that reflects disease severity and inflammatory activity. The strong association observed between the NeuraAAA score and levels of immune cell infiltration further highlights the interconnection between ferroptosis-related pathways and immune dysregulation in AAA pathogenesis.

Several limitations warrant consideration. First, although we integrated multiple GEO datasets and applied batch-effect correction, the analysis is retrospective and based on publicly available transcriptomic data; therefore, residual technical heterogeneity related to platform differences and tissue processing cannot be excluded. Second, subtype identification and pathway interpretation were derived from bulk aortic tissue profiles and may partly reflect inter-sample variation in cellular composition rather than strictly cell-intrinsic regulation. Third, immune infiltration was inferred using CIBERSORT and ESTIMATE; these methods provide useful estimates but require validation with cell-resolved approaches. Fourth, because the analyses are cross-sectional and association based, we cannot infer causality or directionality between ferroptosis-related signals and immune activation.

Future studies should validate the molecular subtypes and the NeuraAAA signature in independent, multicenter cohorts; apply single-cell and spatial profiling to assign key signals to specific vascular and immune cell populations; and incorporate direct measurements of ferroptotic activity in human tissues and experimental models. Mechanistic perturbation studies, including pharmacological and genetic modulation of ferroptosis in vascular cells and relevant AAA models with parallel assessment of immune responses and tissue remodeling, will be necessary to define the causal links within the ferroptosis–immunity axis and to evaluate its therapeutic potential.

## Conclusions

In summary, we systematically evaluated FRG expression in AAA and identified a novel ferroptosis-related molecular classification. Based on neural network machine learning, we constructed a three-gene model capable of effectively identifying distinct ferroptosis-associated molecular subtypes in AAA. External validation of clinical specimens confirmed that IL-6, HMOX-1, and OSM are significantly up-regulated in human AAA tissues. Our study reveals the heterogeneous role of ferroptosis in AAA pathogenesis, demonstrating that ferroptosis-associated subtypes are linked to variations in the immune microenvironment. These findings provide new insights into AAA pathophysiology and suggest potential targets for subtype-specific therapeutic strategies.

## Data Availability

The original contributions presented in the study are included in the article/**Supplementary Material**. Further inquiries can be directed to the corresponding author.
