# Supplementary material for: Artificial neural network-based analysis of ferroptosis-associated molecular subtypes and immunological profiles in abdominal aortic aneurysm
Source: Front Immunol. 2026 Feb 2;17:1721069. doi: 10.3389/fimmu.2026.1721069 (PMC12907547; doi:10.3389/fimmu.2026.1721069)
Supplement: Supplementary file 1 [file DataSheet1.docx]

**Supplementary Tables**

| **Patient**  **ID** | **Sex** | **Age(y)** | **BMI** | **Hypertension** | **Diabetes mellitus** | **History of smoking** | **Dyslipidemia** | **CAD** | **CKD** | **Statin**  **use** | **Symptomatic** | **Diameter of AAA (mm)** |
| --- | --- | --- | --- | --- | --- | --- | --- | --- | --- | --- | --- | --- |
| **P1** | Male | 72 | 20.2 | Yes | No | Yes | Yes | No | No | No | No | 70 |
| **P2** | Male | 66 | 23.0 | No | No | No | Yes | Yes | No | Yes | No | 68 |
| **P3** | Male | 71 | 23.4 | No | No | Yes | No | No | No | No | No | 77 |
| **P4** | Male | 58 | 22.5 | Yes | Yes | Yes | No | No | Yes | No | No | 72 |
| **P5** | Male | 55 | 26.6 | No | Yes | Yes | Yes | No | No | Yes | No | 56 |
| **P6** | Male | 65 | 24.2 | Yes | No | Yes | No | No | No | No | No | 64 |

**Table S1. Demographic and clinical characteristics of patients included in immunofluorescence validation (n=6)**

Abbreviations: BMI, Body Mass Index; CAD, Coronary Artery Disease; CKD, Chronic Kidney Disease, AAA, Abdominal Aortic Aneurysm.

**Table S2. The PAC score (k = 2–9)**

| **k** | **PAC** |
| --- | --- |
| **2** | 0.1899 |
| **3** | 0.3411 |
| **4** | 0.4367 |
| **5** | 0.4113 |
| **6** | 0.3587 |
| **7**  **8**  **9** | 0.2994  0.2752  0.2474 |

Abbreviations: PAC, proportion of ambiguous clustering.
