# Supplementary material for: Artificial neural network-based analysis of ferroptosis-associated molecular subtypes and immunological profiles in abdominal aortic aneurysm
Source: Front Immunol. 2026 Feb 2;17:1721069. doi: 10.3389/fimmu.2026.1721069 (PMC12907547; doi:10.3389/fimmu.2026.1721069)
Supplement: Supplementary file 2 [file DataSheet2.docx]

**Supplementary Figure**


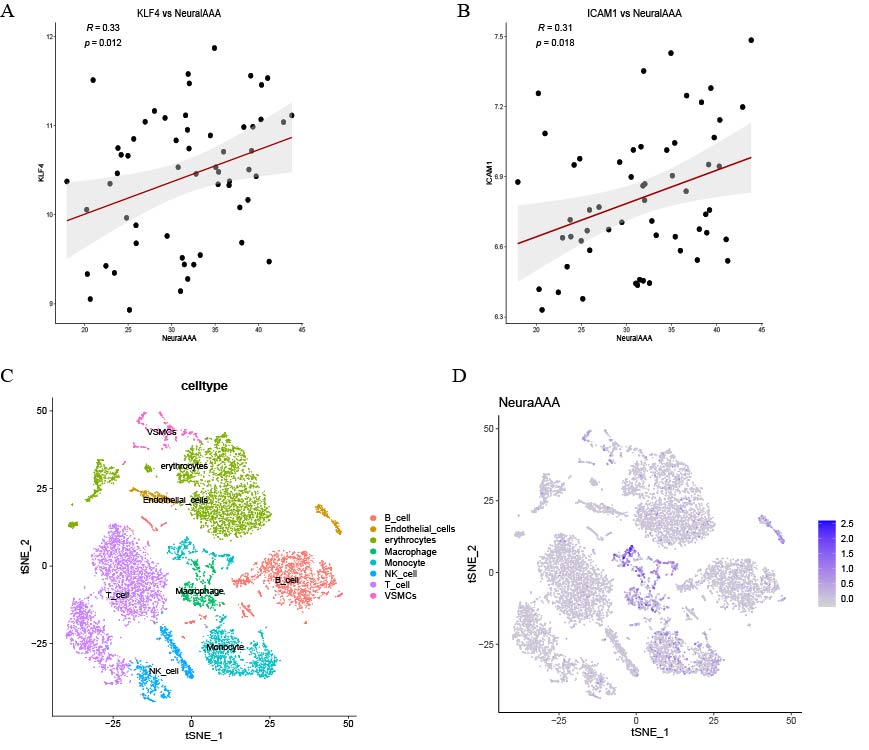


**Supplementary Figure S1. Association of the NeuraAAA score with inflammatory markers and its cellular distribution at the single-cell level**

**(A, B)** Scatter plots showing the correlations between the NeuraAAA score and the expression levels of KLF4 (A) and ICAM1 (B), respectively. Each dot represents one sample. Solid red lines indicate linear regression fits, with shaded areas denoting 95% confidence intervals. Correlation coefficients (R) and corresponding *P* values are shown. **(C)** t-SNE visualization of the integrated single-cell transcriptomic dataset（GSE166676）, with cells colored according to annotated cell types, including B cells, T cells, NK cells, monocytes, macrophages, endothelial cells, erythrocytes, and vascular smooth muscle cells (VSMCs). **(D)** Feature plot showing the distribution of the NeuraAAA score across the t-SNE embedding. Color intensity reflects the relative magnitude of the NeuraAAA module score at the single-cell level. Elevated NeuraAAA scores are predominantly observed in macrophage-enriched regions, indicating cell-type–specific enrichment of the NeuraAAA-associated inflammatory transcriptional program.
